# SNP_tools: A compact tool package for analysis and conversion of genotype data for MS-Excel

**DOI:** 10.1186/1756-0500-2-214

**Published:** 2009-10-23

**Authors:** Bowang Chen, Stefan Wilkening, Marion Drechsel, Kari Hemminki

**Affiliations:** 1 Department of Molecular Genetic Epidemiology, German Cancer Research Center (DKFZ), 69120 Heidelberg, Germany; 2 European Molecular Biology Laboratory (EMBL), 69117 Heidelberg, Germany; 3 Center for Family and Community Medicine, Karolinska Institute, SE-14183 Huddinge, Sweden

## Abstract

**Background:**

Single nucleotide polymorphism (SNP) genotyping is a major activity in biomedical research. Scientists prefer to have a facile access to the results which may require conversions between data formats. First hand SNP data is often entered in or saved in the MS-Excel format, but this software lacks genetic and epidemiological related functions. A general tool to do basic genetic and epidemiological analysis and data conversion for MS-Excel is needed.

**Findings:**

The SNP_tools package is prepared as an add-in for MS-Excel. The code is written in Visual Basic for Application, embedded in the Microsoft Office package. This add-in is an easy to use tool for users with basic computer knowledge (and requirements for basic statistical analysis).

**Conclusion:**

Our implementation for Microsoft Excel 2000-2007 in Microsoft Windows 2000, XP, Vista and Windows 7 beta can handle files in different formats and converts them into other formats. It is a free software.

## Background

The completion of the human genome sequence and the ensued HapMap project has brought a wealth of data on genetic variation in the form of single nucleotide polymorphisms (SNPs) and more recently of copy number variants. These data are accessible through public data bases, such as HapMap [1] or the Cancer Genetic Markers of Susceptibility [2]. As a consequence, SNP genotyping has become a major activity for studies of disease susceptibility and pharmacogenetics. To analyze the data obtained from databases or from own studies, a large number of programs are used, but the first hand SNP data is often entered in or saved in the MS-Excel format. MS-Excel is a good general platform to edit limited amount of data (255 columns and 65,536 rows in MS-Excel 2003) and to do some basic statistical analysis, but it lacks genetic and epidemiological related functions. We developed an MS-Excel add-in, called SNP_tools, to facilitate basic genetic and epidemiological analysis, such as the calculation of odds ratio (OR), confidence interval (CI) p-value, and power.

To further analyze the genotyping data, different programs might be used, for example: Haploview [3], Phase [4], SNPHAP [5], fastPHASE [6], Merlin [7], Plink [8], LdCompare [9] SNPassoc [10], and SPSS (SPSS Inc. Chicago, IL). Since each program has its own requirements for input files there is a need to convert data from one format to another.

## Methods

SNP_tools is implemented in Visual Basic for Application (VBA) in MS-Excel. It can run on MS-Excel 2000-2007 on MS-Windows 2000, XP, Vista and Windows 7 beta. SNP_tools is a free software, which can be redistributed and/or modified under the terms of the GNU Lesser General Public License.

**Figure 1:**
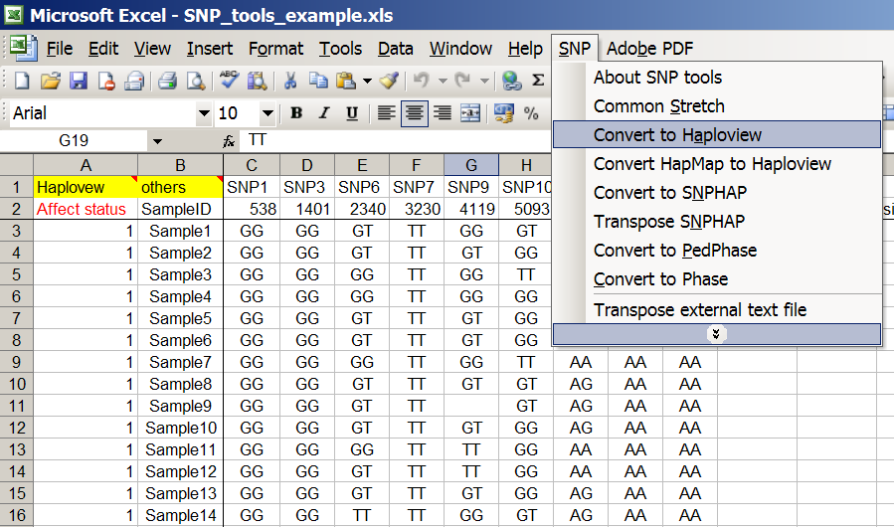
**Main menu of SNP in MS-Excel and the sub-menus of Data conversions**. The different data converters can be started in the new menu item of "SNP" in MS-Excel. Data range and output path can be selected by mouse.

For installation, the file "SNP_tools.xla" has to be copied in the MS Add-In folder: "C:\Documents and Settings\UserID\Application Data\Microsoft\AddIns. MS-Excel has to be opened under Tools > Add-Ins..." Then "SNP_tools" has to be checked. A welcome window will be shown indicating that SNP_tools has been successfully installed. The main menu item "SNP" of SNP_tools should then appear in the tool bar in MS-Excel [see Figure 1]. The SNP_tools requires the existence of Microsoft common dialogue control (Comdlg32.ocx). The missing of 'Comdlg32.ocx' will disable the conversion functions of SNP_tools. Refer to the webpage of SNP_tools to download and install 'Comdlg32.ocx' if the conversion functions can not start.

## Results

### Built-in genetic epidemiology functions

**Figure 2:**
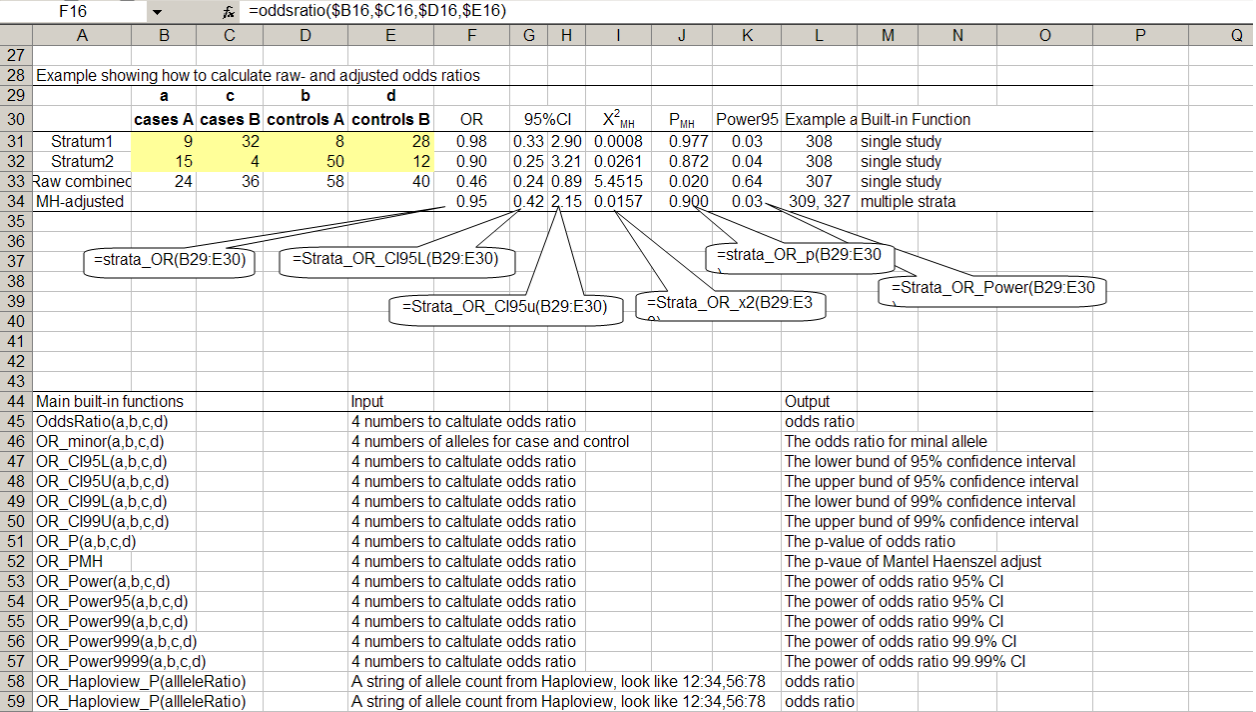
**Built-in genetic epidemiology functions in MS-Excel**. Built-in genetic epidemiological functions can be used by entering as "= function_name(parameters)" in target cell. The parameter can be values or the addresses of cells.

SNP_tools can calculate basic genetic epidemiological parameters in MS-Excel with four numbers of SNP (cases A, cases B, controls A, controls B). The following parameters can be calculated: OR and its CI, chi-square, p-value and power. The range of the CI can be adjusted to 99%, 99.9%, or 99.99%. The Mantel Haenszel stratum-adjusted OR [11] can be obtained by calling the function "strata_OR" with the matrix of cells as input (OR_MH_). After SNP_tools is successfully installed, the new genetic epidemiology functions will start to work [see Figure 2 and Additional file 1].

The value of aberration test of SNPs along the chromosome can be further analysed by functions like "Common Stretch" in section of "Chromosomal Analysis". "Find Hotspot" is a function to look for the chromosomal break point regions by scanning the variation of property variable. "Common Stretch" compares the samples for a common stretch of attribute, such as homozygosity or a no-call stretch. "Compare Haplotypes" is a tool to compare individuals' haplotypes deduced from external programs like SNPHAP or Phase. It creates a matrix of maximum shared length of haplotypes among all individuals and marks the longest common stretches.

### Data Conversion

The "ped file" (*.ped) is a common format in genetic linkage analysis (for example in Haploview) which gives the pedigree and genotype information. The function "Convert to Haploview" in SNP_tools converts genotype data in MS-Excel into an external ped file and the SNP information file (*.info). Users are asked to specify the path and file name to export from MS-Excel. SNP_tools converts the data in the area previously selected with the mouse to specified ped and info files and calls Haploview with the ped and info file names as input files. "Convert to Phase" is a similar tool but saves the data in MS-Excel to an external file in Phase format. "Convert HapMap to Haploview" is a tool which converts genotype data (*.xls) downloaded from the HapMart programme on the HapMap webpage into Haploview format (.ped and .info file). "Convert to SNPHAP" converts data in MS-Excel cells into the data formats (*.nam and *.dat) of SNPHAP, which deduces haplotypes for both populations and individuals. The source code of SNPHAP is written in ANSI C in Linux environment, we have compiled it in Cygwin, and thus SNPHAP could run in MS-Windows (it can be downloaded from the SNP_tools webpage). SNPHAP is a command line program. SNP_tools has a button in graphic user interface to start SNPHAP with necessary input file names (*.nam and *.dat). SNPHAP does haplotyping in the background and saves results in output files. SNP_tools is able to read in these output files back in MS-Excel.

The usages is the same for all data conversion functions: 1) selecting the range of SNP data in MS-Excel, 2) assigning output filenames in common dialogue interface, and 3) clicking the output button to export SNP data into respective data formats. 4) The context button in SNP_tools will call the respective programme to analyse the output files and save results in external files [see Figure 1]. Since the VBA does not accept the space in the file name and the worksheet name, in the case a warning message window will pop up showing the existence of space or other illegal character in the file name or worksheet name.

Different programmes require genotype or haplotype data to be in a specific format (SNPs in columns and Individuals in row or vice versa). The tool "Transpose external text file" allows transposing any text file, whereby the columns are converted into rows. It is especially useful if bigger data sets are handled. MS-Excel has also a transpose tool implemented (Copy, Past Special... and Transpose), however, it is limited to a 256 × 256 matrix (MS-Excel 2003 and earlier versions). Although our transpose tool runs in Excel, the maximal capacity is limited only by the free memory of local computer. It has options to specify the delimiter of input and output files, such as tab, comma, and space in the pull down menu, but users can also specify their own character.

## Conclusion

We have created useful tools for conversion and analysis of genotype files. It has been used in our and other departments with efficiency in daily data management. SNP_tools is freely available for non-commercial use. Users can download it and find the detail usage in .

## Availability and requirements

The SNP_tools for MS-Excel is readily available to any scientist wishing to use it for non-commercial purposes without any restriction. The SNP_tools for MS-Excel can be downloaded for free from the website: . SNP_tools can run on MS-Excel 2000-2007 on MS-Windows 2000, XP, Vista and Windows 7 beta. The prerequisite Microsoft common dialogue control "Comdlg32.ocx" can be also downloaded from the website. Detail user guides and example data for each component can be seen or downloaded in the webpage.

## Competing interests

The authors declare that they have no competing interests.

## Authors' contributions

BC implemented the software and wrote the manuscript. SW tested the software and wrote the manuscript. MD managed the online publication of the software. KH supervised the project, tested the software and contributed to the final draft. All authors read and approved the final manuscript.

## Supplementary Material

Additional file 1 **An example data file of "SNP_tools" for MS-Excel**. The file provides instructions and example data for the use of "SNP_tools" functions. Following the instructions, the new built-in functions will take effect. There is an example data sheet for the conversion of SNP data from users to different external data formats (Haploview, PHASE SNPHAP, etc.). Click here for file
